# Supplementary material for: Prevalence of systemic antibacterial use during pregnancy worldwide: A systematic review
Source: PLoS One. 2024 Sep 6;19(9):e0309710. doi: 10.1371/journal.pone.0309710 (PMC11379220; doi:10.1371/journal.pone.0309710)
Supplement: S1 Table — (PDF) [file pone.0309710.s002.pdf]

S1 Table. Search strategy to identify systemic antibacterial use during pregnancy studies.

**Embase (n=3,276)**	
1	('antibiotic agent'/exp/mj OR 'drug use'/exp OR 'drug utilization'/exp OR 'antibiotic prophylaxis'/exp OR 'antibiotic therapy'/exp OR ('Anti-Bacterial' OR 'Anti-Bacterial' OR 'Antibacterial' OR 'bacteriocid*' OR 'antibiotic*' OR 'Antimicrobial' OR 'prescrib*' OR 'prescription*' OR 'drug utilization' OR 'drug utilisation' OR 'drug use*'):ti,ab,kw)
2	('pregnancy'/exp/mj OR 'pregnant woman'/exp OR 'prenatal care'/de OR ('pregnan*' OR 'Prenatal' OR 'Antenatal' OR 'gestation*'):ti,ab,kw)
3	('epidemiology'/de OR 'epidemiological data'/de OR 'incidence'/de OR 'prevalence'/de OR 'questionnaire'/exp OR 'health survey'/de OR 'observational study'/exp OR 'cohort analysis'/exp OR 'cross-sectional study'/exp OR 'follow up'/exp OR 'longitudinal study'/de OR 'prospective study'/exp OR 'retrospective study'/exp OR epidemiolog*:ti,ab,kw OR observational:ti,ab,kw OR prevalen*:ti,ab,kw OR incidence:ti,ab,kw OR survey*:ti,ab,kw OR questionnaire*:ti,ab,kw OR cohort*:ti,ab,kw OR 'follow-up':ti,ab,kw OR followup:ti,ab,kw OR longitudinal:ti,ab,kw OR prospective:ti,ab,kw OR retrospective:ti,ab,kw OR cross-sectional:ti,ab,kw OR 'population-based':ti,ab,kw)
4	[embase]/lim NOT ([embase]/lim AND [medline]/lim)
5	1 AND 2 AND 3 AND 4 AND 5
**Web of Science (n=10,993)**	
1	TS=("Anti-Bacterial" OR "Anti-Bacterial" OR "Antibacterial" OR "bacteriocid*" OR "antibiotic*" OR "Antimicrobial" OR "prescrib*" OR "prescription*" OR "drug utilization" OR "drug utilisation" OR "drug use*")
2	TS=("pregnan*" OR "Prenatal" OR "Antenatal" OR "gestation*")
3	TS=(epidemiolog* OR observational OR prevalen* OR incidence OR survey* OR questionnaire* OR cohort* OR "follow-up" OR followup OR longitudinal OR prospective OR retrospective OR cross-sectional OR "population-based")
4	1 AND 2 AND 3
**CINAHL (n=2,045)**	
1	(MM "anti bacterial agents" OR MH ("drug prescriptions" OR "drug utilization") OR TI ("Anti-Bacterial" OR "Anti-Bacterial" OR "Antibacterial" OR "bacteriocid*" OR "antibiotic*" OR "Antimicrobial" OR "prescrib*" OR "prescription*" OR "drug utilization" OR "drug utilisation" OR "drug use*") OR AB ("Anti-Bacterial" OR "Anti-Bacterial" OR "Antibacterial" OR "bacteriocid*" OR "antibiotic*" OR "Antimicrobial" OR "prescrib*" OR "prescription*" OR "drug utilization" OR "drug utilisation" OR "drug use*"))
2	MM pregnancy OR MH ("pregnancy trimesters" OR "pregnant women" OR "prenatal care") OR TI ("pregnan*" OR "Prenatal" OR "Antenatal" OR "gestation*") OR AB ("pregnan*" OR "Prenatal" OR "Antenatal" OR "gestation*"))
3	(MH (Prevalence" OR "Incidence" OR "surveys and questionnaires" OR "Health Surveys" OR "Epidemiologic Studies" OR "Cohort Studies" OR "Cross-Sectional Studies") OR TI (epidemiolog* OR observational OR prevalen* OR incidence OR survey* OR questionnaire* OR cohort* OR "follow-up" OR followup OR longitudinal OR prospective OR retrospective OR cross-sectional OR "population-based") OR AB (epidemiolog* OR observational OR prevalen* OR incidence OR survey* OR questionnaire* OR cohort* OR "follow-up" OR followup OR longitudinal OR prospective OR retrospective OR cross-sectional OR "population-based"))
4	1 AND 2 AND 3 AND 4

S1 Table. Continued. Search strategy to identify systemic antibacterial use during pregnancy studies.

**BVS (n=302)**	
1	((mj:D27.505.954.122.085* AND sh:("therapeutic use" OR "administration and dosage")) OR mh:(E02.319.307* OR N04.452.706.477*) OR ti:("Anti-Bacterial" OR "Anti-Bacterial" OR "Antibacterial" OR "bacteriocid*" OR "antibiotic*" OR "Antimicrobial" OR "prescrib*" OR "prescription*" OR "drug utilization" OR "drug utilisation" OR "drug use" OR "Anti-Bacteriano" OR "Antibacteriano" OR "Antimicobacteriano" OR "Anti-micobacteriano" OR "Bactericida" OR "Prescric*" OR "Prescrev*" OR "Prescripcion*" OR "Prescrib*" OR "Uso de Medicamentos" OR "Utilización de Medicamentos" OR "Uso de Fármacos" OR "uso de los medicamentos" OR "utilización de fármacos") OR ab:("Anti-Bacterial" OR "Anti-Bacterial" OR "Antibacterial" OR "bacteriocid*" OR "antibiotic*" OR "Antimicrobial" OR "prescrib*" OR "prescription*" OR "drug utilization" OR "drug utilisation" OR "drug use" OR "Anti-Bacteriano" OR "Antibacteriano" OR "Antimicobacteriano" OR "Anti-micobacteriano" OR "Bactericida" OR "Prescric*" OR "Prescrev*" OR "Prescripcion*" OR "Prescrib*" OR "Uso de Medicamentos" OR "Utilización de Medicamentos" OR "Uso de Fármacos" OR "uso de los medicamentos" OR "utilización de fármacos"))
2	mj:G08.686.784.769 OR mh:(G08.686.707* OR M01.975.807 OR E02.760.786*) OR ti:("pregnan*" OR "Prenatal*" OR "Antenatal" OR "gestation*" OR "Gravidez" OR "Gravida*" OR "Embaraz*" OR "Gestac*" OR "Gestante*" OR "Pré-Natal") OR ab:("pregnan*" OR "Prenatal*" OR "Antenatal" OR "gestation*" OR "Gravidez" OR "Gravida*" OR "Embaraz*" OR "Gestac*" OR "Gestante*" OR "Pré-Natal"))
3	(mh:(prevalence OR incidence OR "surveys and questionnaires" OR "health surveys" OR "epidemiologic Studies" OR E05.318.372.500.500* OR E05.318.372.500.750* OR "cross-sectional studies") OR sh:(epidemiology) OR ti:(epidemiolog* OR observational OR observacional* OR prevalence OR prevalencia OR incidence OR incidencia OR survey* OR encuesta* OR inquerito* OR questionnaire* OR cuestionario* OR questionario* OR cohort* OR coorte* OR "follow up" OR followup OR seguimiento* OR seguimento* OR longitudina* OR prospectiv* OR retrospectiv* OR "cross sectional" OR transversa*) OR ab:(epidemiolog* OR observational OR observacional* OR prevalence OR prevalencia OR incidence OR incidencia OR survey* OR encuesta* OR inquerito* OR questionnaire* OR cuestionario* OR questionario* OR cohort* OR coorte* OR "follow up" OR followup OR seguimiento* OR seguimento* OR longitudina* OR prospectiv* OR retrospectiv* OR "cross sectional" OR transversa*))
4	(db:("LILACS" OR "IBECS" OR "BDENF" OR "CUMED" OR "BBO" OR "INDEXPSI" OR "SES-SP" OR "BINACIS" OR "BDNPAR" OR "LIPECS" OR "MTYCI" OR "colecionaSUS"))
5	1 AND 2 AND 3 AND 4 AND 5
**Scielo (n=96)**	
1	("Anti-Bacterial" OR "Anti-Bacterial" OR "Antibacterial" OR "bacteriocid*" OR "antibiotic*" OR "Antimicrobial" OR "prescrib*" OR "prescription*" OR "drug utilization" OR "drug utilisation" OR "drug use" OR "Anti-Bacteriano" OR "Antibacteriano" OR "Antimicobacteriano" OR "Anti-micobacteriano" OR "Bactericida" OR "Prescric*" OR "Prescrev*" OR "Prescripcion*" OR "Prescrib*" OR "Uso de Medicamentos" OR "Utilización de Medicamentos" OR "Uso de Fármacos" OR "uso de los medicamentos" OR "utilización de fármacos")
2	("pregnan*" OR "Prenatal*" OR "Antenatal" OR "gestation*" OR "Gravidez" OR "Gravida*" OR "Embaraz*" OR "Gestac*" OR "Gestante*" OR "Pré-Natal")
3	(epidemiolog* OR observational OR observacional* OR prevalence OR prevalencia OR incidence OR incidencia OR survey* OR encuesta* OR inquerito* OR questionnaire* OR cuestionario* OR questionario* OR cohort* OR coorte* OR "follow up" OR followup OR seguimiento* OR seguimento* OR longitudina* OR prospectiv* OR retrospectiv* OR "cross sectional" OR transversa*)
4	1 AND 2 AND 3 AND 4
