# Supplementary material for: Prevalence of systemic antibacterial use during pregnancy worldwide: A systematic review
Source: PLoS One. 2024 Sep 6;19(9):e0309710. doi: 10.1371/journal.pone.0309710 (PMC11379220; doi:10.1371/journal.pone.0309710)
Supplement: S2 Table — (PDF) [file pone.0309710.s003.pdf]

S2 Table. Characteristics of studies included in the systematic review.

Author, year	Design	Country	Data assessment	Denominator type	Maternal schooling	Maternal age (N or %)	Cesarean (N or %)	Denominator (N)	Antibacterial use during pregnancy (n)
Cassidy-Bushrow, 2018	Cohort	United States	prescriptions database	Mother-child dyad				527	303
Stokholm, 2013	Cohort	Denmark	prescriptions database	Mother-child dyad	Low: 75(11%)<br>Medium: 406(58%)<br>High: 214(31%)	Mean(SD): 32.3(4.4)		738	708
Laursen, 2020	Cross-sectional	Denmark	Questionnaires	Pregnant women	School only: 8639(15.3%)<br>Secondary school: 18488(32.8%)<br>Higher education: 17431(30.9%)<br>University master or PhD: 8201(14.6%)	<25: 6424(11.6%)<br>25-29: 16,638 (30.1%)<br>30-34: 19,588 (35.5%)<br>>34: 12,583 (22.8%)		753	178
Pisa, 2015	Cohort	Italy	prescriptions database and self-administered questionnaires	Pregnant women	<High school: 139(18.1%)<br>High school: 364(47.5%)<br>University: 262 (34.2%)	25: 42(5.5%)<br>25-29: 111(14.5%)<br>30-34: 327(42.6%)<br>35-39: 229(29.8%)<br>40+: 58(7.6%)		767	20
Romanese, 2018	Cohort	Italy	Self-administered questionnaire	Pregnant women	Less than high school: 224(29%)<br>High school: 348 (45%)<br>University: 180(23%)	<25: 42(5.5%)<br>25-29: 111 (14.5%)<br>30-34: 229(29.9%)<br>≥40: 58(7.6%)		767	20
Leke, 2018	Cross-sectional	Cameroon	questionnaires	Pregnant women	Never went to school: 19(2.4%)<br>Primary: 205(25.8%)<br>Secondary: 329(41.4%)<br>High School: 129 (16.2%)<br>University/professional: 113(14.2%)	13-17: 41(5.2%)<br>18-25: 380(47.8%)<br>26-35: 335(42.1%)<br>36-45: 39(4.9%)		795	165

S2 Table. Continued. Characteristics of studies included in the systematic review.

Author, year	Design	Country	Data assessment	Denominator type	Maternal schooling	Maternal age (N or %)	Cesarean (N or %)	Denominator (N)	Antibacterial use during pregnancy (n)
Metzler, 2019	Cohort	Austria, Finland, France, Germany and Switzerland	Questionnaires	Mother-child dyad	Low: 202 (17.8%)<br>Mid: 487 (43%)<br>High: 444 (39.2%)		192 (17.7%)	1080	288
Costa, 2017	Cross-sectional	Brazil	Questionnaires	Pregnant women	≤8: 539(46.7%)<br>9-11: 487(44.8%)<br>>11: 62(5.7%)	18-24: 509(46.7%)<br>25-29: 290(26.6%)<br>30-45: 292(26.8%)		1091	34
Kelderer, 2022	Cohort	Sweden	questionnaires	Mother-child dyad		30.9 (4.4)		1387	114
Mueller, 2017	Cohort	United States	prescriptions database	Pregnant women	College graduate<br>No: 744 (38.8)<br>Yes: 1360 (64.6)	Mean (SD): 31.8 (5.2)	498 (23%)	2128	643
Bonassi, 1994	Cross-sectional	Italy	questionnaires	Pregnant women	Years of education<br>≤8: 1611(52%)<br>>8 years: 1478(48%)	<26: 894 (29%)<br>26-30: 1212(39%)<br>>30: 945(31%)		3112	218
Ceulemans, 2022	Cross-sectional	Ireland, Norway, Switzerland, Netherlands, United Kingdom	Online Questionnaires	Pregnant women				3339	88
Guimaraes, 2021	Cross-sectional	Brazil	Questionnaires	Pregnant women	2004 cohort / 2015 cohort<br>0-4: 647 / 387<br>5-8: 1711 / 1084<br>9-11: 1372 / 1442<br>12+: 417 / 1305	2004 cohort: ≤19: 796<br>20-29: 2085<br>≥30: 1306<br>2015 cohort: ≤19: 618<br>20-29: 1995<br>≥30: 1605		2004 cohort: 4189<br>2015 cohort: 4219	2004 cohort: 1753<br>2015 cohort: 1652

S2 Table. Continued. Characteristics of studies included in the systematic review.

Author, year	Design	Country	Data assessment	Denominator type	Maternal schooling	Maternal age (N or %)	Cesarean (N or %)	Denominator (N)	Antibacterial use during pregnancy (n)
Bakker, 2006	Cohort	Netherlands	prescriptions database	Pregnant women		29.6 (range: 15-49 years)		5412	Use by trimesters:<br>1st: 340<br>2nd: 395<br>3rd: 476
Hu, 2021	Cross-sectional	Canada	Questionnaires	Mother-child dyad			2709 (60%)	4500	468
Elfrink, 2013	Cohort	Netherlands	prescriptions database	Pregnancies		30.6 (5.2)		5613	569
Fossum, 2018	Cohort	Norway	prescriptions database	Mother-child dyad		Mean(SD): 30.48(4.9)		7747	1948
Zhang, 2019	Cross-sectional	China	prescriptions database	Pregnant women				7946	Use by trimesters:<br>1st: 1922<br>2nd: 2089<br>3rd: 1962
Marild, 2014	Cohort	Sweden	Questionnaires	Mother-child dyad				8729	1836
Valent, 2014	Cross-sectional	Italy	prescriptions database	Pregnant women		Mean: 32		9196	2279
Haas, 2018	Cohort	United States	Questionnaires	Pregnant women		Mean (SD): 27 (5,6)		9546	2439
Mor, 2015	Cross-sectional	Denmark	prescriptions database			≤20: 302(3%)<br>21-34: 8413(85%)<br>≥35: 1171(12%)	963 (10%)	9886	3280
Lin, 2020	Cohort	China	prescriptions database	Mother-child dyad	≤9: 1787<br>>9: 6795		3507	10163	4909
Cunha, 2021	Cohort	United Kingdom	prescriptions database	Mother-child dyad		Mean (SD): 27.5 (5.6)		12476	4579
Mission, 2019	Cohort	United States	prescriptions database	Pregnant women		Mean (SD): 28.1(+-5.9)		12551	3991

S2 Table. Continued. Characteristics of studies included in the systematic review.

Author, year	Design	Country	Data assessment	Denominator type	Maternal schooling	Maternal age (N or %)	Cesarean (N or %)	Denominator (N)	Antibacterial use during pregnancy (n)
Headley, 2004	Cohort	England	Self-administerd questionnaire	Pregnant women	CSE(lowest): 2494<br>Vocational: 1216<br>O level: 4275<br>A level: 2771<br>Degree(highest): 1587			13.548	Use by trimesters:<br>1st: 1085<br>2nd: 978/13194<br>3rd: 719/12421
Araujo, 2021	Cross-sectional	France	prescriptions database	Pregnancies				18279	6219
de Jonge, 2013	Cross-sectional	Netherland s	prescriptions database	Pregnancies				18873	3916
Lee, 2016	Cross-sectional	Taiwan	prescriptions database	Pregnancies				19464	6554
Olesen, 2006	Cohort	Denmark	prescriptions database	Pregnant women				19874	6730
Demailly, 2017	Cohort	France	Reimbursed purchases of prescribed medicines	Pregnancies		Mean: 30(range:27-34)		28491	14503
Berard, 2019	Cohort	France	Reimbursed purchases of prescribed medicines	Pregnancies		36.1 (9.2)		32408	11534
Uldbjerg, 2021	Cohort	Denmark	telephone and online questionnaires	Pregnant women		≤19: 0.2%<br>20-24: 6.7%<br>25-29: 38.4%<br>30-34: 38.8%<br>≥35: 16%	14%	32651	5522
Chu, 2015	Cohort	United States	Questionnaires	Mother-child dyad	≤9: 11121<br>10-12: 23679<br>>12: 4509	<20: 9365<br>20-29: 22552<br>30-39: 7257<br>≥40: 733	2057	39907	10534

S2 Table. Continued. Characteristics of studies included in the systematic review.

Author, year	Design	Country	Data assessment	Denominator type	Maternal schooling	Maternal age (N or %)	Cesarean (N or %)	Denominator (N)	Antibacterial use during pregnancy (n)
Amann, 2006	Cross-sectional	Germany	prescriptions database	Pregnant women		Mean (SD): 31.7 (4.5)		41293	8142
Wang, 2018	Cohort	United States	Self-administered questionnaire	Pregnant women	≤9: 12277<br>10-12: 25555<br>>12: 4750	<20: 10230<br>20-34: 29665<br>≥35: 3386		43332	10908
Jess, 2019	Cohort	Denmark	prescriptions database	Mother-child dyad			11581/81229	43365	10754
Gerbier, 2021	Cross-sectional	Switzerland	Reimbursed purchases of prescribed medicines	Pregnancies		Mean(SD): 32(5.1)	33.7%	45251	12016
Heerman, 2019	Cohort	United States	prescriptions database	Mother-child dyad				53320	15516
Rantala, 2022	Cohort	Norway	prescriptions database and questionnaires	Pregnant women				417548	118287
Tomar, 2022	Cohort	Denmark	prescriptions database	Pregnant women		<20: 269<br>20-24.99: 5292<br>25-29.99: 24520<br>30-34.99: 23615<br>≥35: 9604		63300	11053
Rozanska, 2021	Cohort	Poland	prescriptions database	Pregnant women			29551	67917	16059
Hardy, 2006	Cohort	United Kingdom	prescriptions database	Pregnancies				81975	9489
Yoshida, 2018	Cohort	Japan	prescriptions database	Mother-child dyad				83470	20630
Turi, 2021	Cohort	United States	prescriptions database	Mother-child dyad		Median: 22 (interquartile interval=19-26)		84214	54147

S2 Table. Continued. Characteristics of studies included in the systematic review.

Author, year	Design	Country	Data assessment	Denominator type	Maternal schooling	Maternal age (N or %)	Cesarean (N or %)	Denominator (N)	Antibacterial use during pregnancy (n)
Marild, 2017	Cohort	Norway	prescriptions database	Mother-child dyad				84274	13134
Sassonker-Joseph, 2021	Cohort	Israel	Reimbursed purchases of prescribed medicines	Mother-child dyad				88899	36662
Jacob, 2017	Cohort	Germany	prescriptions database	Pregnant women		Mean(SD): 29.8 (5.3)		90312	13260
Nishigori, 2017	Cohort	Japan	Questionnaires	Pregnant women	Junior/highschool: 35109(36%)<br>Technical/junior college: 40517(41.6%)<br>University/graduate school: 20816(21.4%)	30.8 (5.0)		97464	11638
Petersen, 2010	Cohort	United Kingdom	prescriptions database	Pregnant women		30.5 years (interquartile range: 26-34)		114999	37468
Leong, 2020	Cohort	New Zealand	prescriptions database	Mother-child dyad		<20: 10 296 (6.8%)<br>20-24: 26 928 (17.8%)<br>25-29: 37 377 (24.7%)<br>30-34: 42 834 (28.3%)<br>35-39: 27 693 (18.3%)<br>≥40: 6225 (4.1%)		132852	54078

S2 Table. Continued. Characteristics of studies included in the systematic review.

Author, year	Design	Country	Data assessment	Denominator type	Maternal schooling	Maternal age (N or %)	Cesarean (N or %)	Denominator (N)	Antibacterial use during pregnancy (n)
Daw, 2012	Cohort	Canada	prescriptions database	Pregnancies		Mean: 30.2		163082	49805
Snyder, 2021	Cross-sectional	United States	prescriptions database	Pregnant women	<12 years: 69220(41%)<br>12 years: 73261(44%)<br>>12 years: 25527(15%)	22 years (interquartile range: 19-26)	37802 (22%)	168354	105139
Hamad, 2020	Cohort	Canada	prescriptions database	Mother-child dyad			37867 (40.6%)	187605	70554
Meeraus, 2015	Cohort	United Kingdom	prescriptions database	Pregnant women		15-19: 6.9%<br>20-24: 18.1%<br>25-29: 26.9%<br>30-34: 29.1%<br>35-39: 15.8%<br>40-50: 3.3%		195909	64623
Loewen, 2018	Cohort	Canada	prescriptions database	Mother-child dyad		Mean (SD): 27.6 (5.9)	41248 (19.3)	213661	78522
Koebnick, 2019	Cohort	United States	Record of drug dispenses and administration	Mother-child dyad				223431	42511
Ye, 2019	Cohort	Canada	prescriptions database	Mother-child dyad		<20: 11 294 (11.4%)<br>20-24: 4 882 (25.1%)<br>25-29: 28 967 (29.3%)<br>30-34: 22 806 (23.0%)<br>35+: 1 048 (11.2%)		262116	98997
Berard, 2014	Cohort	Canada	prescriptions database	Pregnancies	Secondary 1&2: 8.8%<br>Secondary 3,4&5: 38.7%<br>College: 23.5%<br>University: 15.2%	Mean(SD): 27.8(5.6)		289688	75609

S2 Table. Continued. Characteristics of studies included in the systematic review.

Author, year	Design	Country	Data assessment	Denominator type	Maternal schooling	Maternal age (N or %)	Cesarean (N or %)	Denominator (N)	Antibacterial use during pregnancy (n)
Momen, 2021	Cohort	Denmark	prescriptions database	Pregnant women	Elementary school: 31122(20.9%)<br>Above elementary school: 113206(76.1%)<br>Unkown: 4454(3.0%)	<25: 27156(10.5%)<br>25-34: 181006(69.9%)<br>≥35: 50860(19.6%)	25955 (17.4%)	407804	148782
Rantala, 2022	Cohort	Norway	prescriptions database and questionnaires	Pregnant women				417548	118287
Nguyen, 2022	Cohort	Sweden	prescriptions database	Pregnant women		≤25: 29.3%<br>26-30: 37.4%<br>≥31: 33.3%		483706	98963
Ortqvist, 2014	Cohort	Sweden	prescriptions database	Mother-child dyad		<19: 7746<br>19-24: 61960<br>25-29: 140790<br>30-34: 173923<br>≥35: 109366	88503	493785	101135
Lovern, 2022	Cohort	Norway	prescriptions database	Mother-child dyad				539390	146832
Stokholm, 2014	Cohort	Denmark	prescriptions database	Mother-child dyad				545394	289348
Miller, 2013	Cohort	Denmark	prescriptions database	Mother-child dyad		<20: 3519(2%)<br>20-24: 25392(15%)<br>25-29: 61290(35%)<br>30-34: 58011(34%)<br>≥35: 24667(14%)		551518	172879
Artama, 2011	Cross-sectional	Finland	Reimbursed purchases of prescribed medicines	Pregnancies				622672	168121

S2 Table. Continued. Characteristics of studies included in the systematic review.

Author, year	Design	Country	Data assessment	Denominator type	Maternal schooling	Maternal age (N or %)	Cesarean (N or %)	Denominator (N)	Antibacterial use during pregnancy (n)
Miller, 2013	Cohort	Denmark	prescriptions database	Mother-child dyad		<20: 3519(2%)<br>20-24: 25392(15%)<br>25-29: 61290(35%)<br>30-34: 58011(34%)<br>≥35: 24667(14%)		551518	172879
Artama, 2011	Cross-sectional	Finland	Reimbursed purchases of prescribed medicines	Pregnancies				622672	168121
Engeland, 2018	Cohort	Norway	prescriptions database			<20: 12462 (2%)<br>20-34: 503586 (78.9%)<br>35-44: 121619 (19%)<br>≥45: 865(0.1%)		638532	178150
Trinh, 2021	Cross-sectional	Norway	prescriptions database	Pregnancies		≤24: 102308 (15.7%)<br>25-29: 206141(31.6%)<br>30-34: 216922(33.2%)<br>≥35: 127687(19.5%)		653058	180516
Broe, 2014	Cohort	Denmark	prescriptions database	Pregnancies	Years of education:<br>7-10: 125667<br>11-12: 280456<br>13+: 275872	<20: 16134<br>20-29: 346923<br>30-39: 321189<br>40+: 12052		696298	232889
Mubanga, 2021	Cohort	Sweden	prescriptions database	Mother-child dyad	Primary: 18619 (12%)<br>Secondary: 59663 (39%)<br>Tertiary or higher: 73522 (49%)		28736 (19.2%)	722767	153407
Cantarutti, 2021	Cohort	Italy	prescriptions database	Pregnant women				773237	208935

S2 Table. Continued. Characteristics of studies included in the systematic review.

Author, year	Design	Country	Data assessment	Denominator type	Maternal schooling	Maternal age (N or %)	Cesarean (N or %)	Denominator (N)	Antibacterial use during pregnancy (n)
Miller, 2018	Cohort	Denmark	prescriptions database	Mother-child dyad				776657	141359
Mølgaard-Nielsen, 2012	Cohort	Denmark	prescriptions database	Mother-child dyad				806011	549880
Donald, 2020	Cohort	New Zealand	prescriptions database	Pregnancies		15-19: 89854(10.3%)<br>20-29: 385554(44.1%)<br>30-39: 361471(41.3%)<br>40-49: 38005(4.3%)		874884	227470
Ingstrup, 2017	Cross-sectional	Denmark	prescriptions database	Pregnancies		Mean(SD): 30.1(4.9)		981391	331710
Lavebratt, 2019	Cohort	Finland	Record of purchases during pregnancy	Mother-child dyad				990098	254455
Momen, 2015	Cohort	Denmark and Sweden	prescriptions database	Mother-child dyad	Years of education<br>≤9: 237123(16%)<br>10-14: 698079(48%)<br>≥15: 489553(33%)	≤24: 189 614 (13%)<br>25-34: 964 064 (67%)<br>35-44: 286 452 (20%)<br>≥45: 1984 (<1)		1442114	506194
Zhao, 2021	Cross-sectional	China	prescriptions database	Ambulatory care visits of pregnant women				4574961	92514
