# Supplementary material for: Prevalence of systemic antibacterial use during pregnancy worldwide: A systematic review
Source: PLoS One. 2024 Sep 6;19(9):e0309710. doi: 10.1371/journal.pone.0309710 (PMC11379220; doi:10.1371/journal.pone.0309710)
Supplement: S3 Table — (PDF) [file pone.0309710.s004.pdf]

S3 Table. Proportion of antibacterial subgroups of studies included in the systematic review.

**Author, year**	Antibacterial subgroups N (%)					
	Beta-lactam (including cephalosporins)	Sulphonamides and trimethoprim	Macrolides, lincosamides and streptogramins	Tetracyclines	Quinolones	Nitrofuran derivatives and imidazole
Cantarutti, 2021	92027 (13.2)	-	24763 (3.6)	-	6352 (0.9)	-
Cassidy-Bushrow, 2018	276 (52.3)	-	62 (11.7)	-	-	72 (13.6)
Chu, 2015	6823 (64.7)	3879 (36.8)	-	530 (5.0)	-	379 (3.6)
Costa, 2017	33 (3.1)	-	-	-	-	5 (0.5)
Engeland, 2018	151970 (23.8)	10216 (1.6)	17878 (2.8)	2554 (0.4)	-	-
Guimaraes, 2021	2004 cohort: 1112 (47.2)<br>2015 cohort: 584 (27.7)	2004 cohort: 28 (1.2)<br>2015 cohort: 7 (0.3)	2004 cohort: 14 (0.6)<br>2015 cohort: 42 (2.0)	-	2004 cohort: 66 (2.8)<br>2015 cohort: 21 (1.0)	2004 cohort: 38 (1.6)<br>2015 cohort: 358 (17.0)
Haas, 2018	1079 (11.3)	97 (1.0)	431 (4.5)	-	-	656 (6.9)
Jacob, 2017	4826 (36.4)	-	1657 (12.5)	-	-	-
Jess, 2019	7969 (19.6)	1571 (4.6)	931 (2.8)	-	-	-
Lee, 2016	5324 (1.9)	27 (0.01)	488 (0.17)	340 (0.12)	523 (0.19)	-
Leong, 2020	41449 (31.2)	41183 (3.1)	93527 (7.4)	-	-	-
Loewen, 2018	52598 (24.6)	5615 (2.6)	15120 (7.1)	16074 (7.5)	-	-
Meeraus, 2015	27577 (66.8)	1375 (3.3)	2749 (6.7)	-	-	802 (1.0)
Miller, 2013	135925 (78.6)	37991 (22.0)	20451 (11.8)	-		6926 (4.0)
Miller, 2018	112787 (20.4)	29956 (5.4)	14843 (2.7)	1153 (0.2)	613 (0.1)	-

S3 Table. Continued. Proportion of antibacterial subgroups of studies included in the systematic review.

**Author, year**	Antibacterial subgroups N (%)					
	Beta-lactam (including cephalosporins)	Sulphonamides and trimethoprim	Macrolides, lincosamides and streptogramins	Tetracyclines	Quinolones	Nitrofuran derivatives and imidazole
Mission, 2019	1110 (8.9)	-	-	-	-	-
Mor, 2015	2102 (21.2)	330 (3.3)	6 (0.06)	19 (0.2)	-	-
Mueller, 2017	514 (44.7)	-	23 (2.0)	42 (3.7)	-	191 (16.6)
Nguyen, 2022	69540 (70.2)	1788 (1.8)	3767 (3.8)	2984 (3.0)	1365 (1.4)	-
Petersen, 2010	24894 (21.6)	1745 (1.5)	3897 (3.4)	407 (0.4)	219 (0.2)	1436 (1.2)
Sassonker-Joseph, 2021	18906 (21.3)	337 (0.4)	1779 (2.0)	1060 (1.2)	610 (0.7)	-
Wang, 2018	6189 (56.7)	4339 (39.8)	202 (1.9)	1494 (13.7)	-	522 (4.8)
Yoshida, 2018	11557 (14.5)	-	5721 (7.2)	-	-	-
Zhang, 2019	770 (9.7)	-	611 (7.7)	-	-	-

Note: The table indicates studies with data on antibacterial subgroups (N=24).
