# Supplementary material for: Prevalence of systemic antibacterial use during pregnancy worldwide: A systematic review
Source: PLoS One. 2024 Sep 6;19(9):e0309710. doi: 10.1371/journal.pone.0309710 (PMC11379220; doi:10.1371/journal.pone.0309710)
Supplement: S4 Table — (PDF) [file pone.0309710.s005.pdf]

S4 Table. Quality assessment of included studies (n=79) using the Joanna Briggs Institute critical appraisal tools (adapted version).

Author, year.	Q1	Q2	Q3	Q4	Q5	Q6	Q7	Q8	Q9
Amann, 2006	Yes	Yes	Yes	Yes	No	Yes	Yes	U	U
Araujo, 2021	Yes	Yes	Yes	U	U	Yes	Yes	U	No
Artama, 2011	Yes	Yes	Yes	Yes	Yes	Yes	Yes	U	U
Bakker, 2006	Yes	Yes	Yes	No	U	Yes	Yes	U	Yes
Berard, 2014	Yes	Yes	Yes	Yes	Yes	Yes	Yes	U	U
Berard, 2019	Yes	Yes	Yes	Yes	Yes	Yes	Yes	U	U
Bonassi, 1994	Yes	Yes	U	Yes	U	U	Yes	U	No
Broe, 2014	Yes	Yes	Yes	Yes	Yes	Yes	Yes	U	Yes
Cantarutti, 2021	Yes	Yes	U	Yes	Yes	Yes	Yes	U	Yes
Cassidy-Bushrow, 2018	Yes	Yes	Yes	Yes	Yes	Yes	U	NA	Yes
Ceulemans, 2022	Yes	U	U	Yes	U	Yes	Yes	U	No
Chu, 2015	Yes	Yes	U	Yes	Yes	U	U	NA	Yes
Costa, 2017	Yes	Yes	Yes	Yes	Yes	Yes	Yes	No	U
Cunha, 2021	Yes	Yes	Yes	Yes	U	Yes	U	NA	Yes
Daw, 2012	Yes	Yes	Yes	U	U	Yes	Yes	U	U
de Jonge, 2013	Yes	Yes	Yes	U	Yes	Yes	Yes	U	No
Demailly, 2017	Yes	Yes	Yes	U	Yes	Yes	Yes	U	Yes
Donald, 2020	Yes	Yes	Yes	Yes	Yes	U	Yes	U	U
Elfrink, 2013	Yes	Yes	Yes	U	Yes	Yes	U	NA	Yes
Engeland, 2018	Yes	Yes	Yes	Yes	U	Yes	Yes	U	U
Fossum, 2018	Yes	Yes	Yes	U	U	U	Yes	NA	U
Gerbier, 2021	Yes	Yes	Yes	U	Yes	Yes	Yes	Yes	U
Guimaraes, 2021	Yes	Yes	U	Yes	Yes	Yes	Yes	Yes	Yes
Haas, 2018	Yes	Yes	Yes	Yes	U	U	U	U	U
Hamad, 2020	Yes	Yes	Yes	Yes	U	Yes	Yes	NA	U
Hardy, 2006	Yes	Yes	Yes	Yes	Yes	Yes	U	U	U
Headley, 2004	Yes	Yes	U	Yes	U	U	Yes	U	U
Heerman, 2019	Yes	Yes	Yes	Yes	U	U	Yes	NA	U
Hu, 2021	Yes	Yes	Yes	Yes	Yes	U	U	NA	U
Ingstrup, 2017	Yes	Yes	Yes	Yes	U	U	Yes	Yes	U
Jacob, 2017	Yes	Yes	Yes	Yes	U	Yes	Yes	U	U
Jess, 2019	Yes	Yes	Yes	Yes	Yes	U	Yes	NA	Yes
Kelderer, 2022	Yes	Yes	Yes	Yes	Yes	Yes	U	NA	Yes
Koebnick, 2019	Yes	Yes	Yes	Yes	Yes	Yes	U	U	Yes
Laursen, 2020	Yes	Yes	Yes	Yes	U	Yes	Yes	U	Yes
Lavebratt, 2019	Yes	Yes	Yes	Yes	U	Yes	Yes	NA	U
Lee, 2016	Yes	Yes	Yes	U	U	Yes	Yes	U	U
Leke, 2018	Yes	Yes	Yes	Yes	U	Yes	Yes	NA	U
Leong, 2020	Yes	Yes	Yes	Yes	U	Yes	U	U	U
Lin, 2020	Yes	Yes	U	Yes	Yes	U	Yes	NA	U
Loewen, 2018	Yes	Yes	U	Yes	U	Yes	Yes	NA	U
Lovern, 2022	Yes	Yes	Yes	Yes	U	Yes	Yes	NA	U
Marild, 2014	Yes	Yes	Yes	U	Yes	Yes	U	NA	Yes
Marild, 2017	Yes	Yes	Yes	U	U	Yes	Yes	NA	Yes
Meeraus, 2015	Yes	Yes	Yes	Yes	U	Yes	Yes	NA	U
Metzler, 2019	Yes	Yes	U	Yes	U	U	U	NA	U
Miller, 2013	Yes	Yes	Yes	Yes	U	U	Yes	NA	U
Miller, 2018	Yes	Yes	Yes	Yes	U	Yes	Yes	NA	U

S4 Table. Continued. Quality assessment of included studies (n=79) using the Joanna Briggs Institute critical appraisal tools (adapted version).

Mission, 2019	Yes	Yes	Yes	Yes	Yes	Yes	U	NA	U
Mølgaard-Nielsen, 2012	Yes	Yes	Yes	Yes	U	U	Yes	NA	No
Momen, 2015	Yes	Yes	Yes	Yes	U	Yes	Yes	NA	U
Momen, 2021	Yes	Yes	Yes	U	Yes	Yes	Yes	NA	U
Mor, 2015	Yes	Yes	Yes	Yes	U	Yes	U	NA	U
Mubanga, 2021	Yes	Yes	Yes	Yes	U	Yes	Yes	NA	U
Mueller, 2017	Yes	Yes	Yes	Yes	U	Yes	Yes	NA	U
Nguyen, 2022	Yes	Yes	Yes	Yes	U	U	Yes	NA	U
Nishigori, 2017	Yes	Yes	Yes	Yes	Yes	Yes	U	U	U
Olesen, 2006	Yes	Yes	Yes	Yes	U	Yes	Yes	NA	U
Ortqvist, 2014	Yes	Yes	Yes	Yes	U	Yes	Yes	NA	U
Petersen, 2010	Yes	Yes	Yes	U	Yes	Yes	Yes	U	U
Pisa, 2015	Yes	Yes	U	Yes	No	U	Yes	U	U
Rantala, 2022	Yes	Yes	Yes	Yes	Yes	Yes	Yes	NA	U
Romanese, 2018	Yes	U	U	Yes	Yes	Yes	Yes	U	U
Rozanska, 2021	Yes	Yes	Yes	Yes	U	Yes	Yes	U	U
Sassonker-Joseph, 2021	Yes	Yes	U	Yes	U	Yes	Yes	NA	U
Snyder, 2021	Yes	Yes	Yes	Yes	U	Yes	Yes	NA	U
Stephansson, 2011	Yes	Yes	Yes	U	U	U	Yes	U	No
Stokholm, 2013	Yes	Yes	U	Yes	U	Yes	Yes	NA	U
Stokholm, 2014	Yes	Yes	Yes	U	Yes	U	U	No	NA
Tomar, 2022	Yes	Yes	Yes	Yes	Yes	U	U	NA	Yes
Trinh, 2021	Yes	Yes	Yes	Yes	Yes	Yes	Yes	U	Yes
Turi, 2021	Yes	Yes	Yes	Yes	U	U	U	NA	U
Uldbjerg, 2021	Yes	Yes	Yes	Yes	Yes	U	U	NA	No
Valent, 2014	Yes	Yes	Yes	U	U	Yes	Yes	U	U
Wang, 2018	Yes	Yes	Yes	Yes	U	Yes	Yes	NA	Yes
Ye, 2019	Yes	Yes	U	U	U	Yes	Yes	NA	U
Yoshida, 2018	Yes	Yes	Yes	U	Yes	Yes	Yes	NA	Yes
Zhang, 2019	Yes	Yes	Yes	U	Yes	U	Yes	U	Yes
Zhao, 2021	Yes	Yes	Yes	Yes	Yes	Yes	Yes	Yes	Yes

NA: not applicable, U: unclear.
