# Supplementary material for: Prevalence of systemic antibacterial use during pregnancy worldwide: A systematic review
Source: PLoS One. 2024 Sep 6;19(9):e0309710. doi: 10.1371/journal.pone.0309710 (PMC11379220; doi:10.1371/journal.pone.0309710)
Supplement: S5 Table — (PDF) [file pone.0309710.s006.pdf]

Supplementary Table 5. Data extraction summary and eligibility confirmation of included studies (n=79).

Author, year.	Date of extraction	Name of data extractors	Eligibility confirmation		
			Study design: population-based cross sectional or cohort	Population: pregnant women	% of systemic antibiotics in the first, second or third trimester of pregnancy (at least)
Amann, 2006	February-2023	FSG	yes	yes	yes
Araujo, 2021	March-2023	MPS	yes	yes	yes
Artama, 2011	March-2023	MPS	yes	yes	yes
Bakker, 2006	February-2023	FSG	yes	yes	yes
Berard, 2014	March-2023	TSD	yes	yes	yes
Berard, 2019	March-2023	TSD	yes	yes	yes
Bonassi, 1994	February-2023	FSG	yes	yes	yes
Broe, 2014	February-2023	FSG	yes	yes	yes
Cantarutti, 2021	March-2023	TSD	yes	yes	yes
Cassidy-Bushrow, 2018	March-2023	TSD	yes	yes	yes
Ceulemans, 2022	March-2023	TSD	yes	yes	yes
Chu, 2015	March-2023	MPS	yes	yes	yes
Costa, 2017	March-2023	TSD	yes	yes	yes
Cunha, 2021	February-2023	FSG	yes	yes	yes
Daw, 2012	March-2023	TSD	yes	yes	yes
de Jonge, 2013	February-2023	FSG	yes	yes	yes
Demailly, 2017	March-2023	TSD	yes	yes	yes
Donald, 2020	March-2023	MPS	yes	yes	yes
Elfrink, 2013	March-2023	TSD	yes	yes	yes
Engeland, 2018	March-2023	MPS	yes	yes	yes
Fossum, 2018	March-2023	TSD	yes	yes	yes
Gerbier, 2021	March-2023	TSD	yes	yes	yes
Guimaraes, 2021	March-2023	TSD	yes	yes	yes
Haas, 2018	March-2023	TSD	yes	yes	yes
Hamad, 2020	March-2023	MPS	yes	yes	yes
Hardy, 2006	March-2023	MPS	yes	yes	yes
Headley, 2004	February-2023	FSG	yes	yes	yes
Heerman, 2019	March-2023	MPS	yes	yes	yes
Hu, 2021	March-2023	MPS	yes	yes	yes
Ingstrup, 2017	March-2023	TSD	yes	yes	yes
Jacob, 2017	March-2023	TSD	yes	yes	yes
Jess, 2019	March-2023	TSD	yes	yes	yes
Kelderer, 2022	February-2023	FSG	yes	yes	yes
Koebnick, 2019	March-2023	MPS	yes	yes	yes
Laursen, 2020	March-2023	MPS	yes	yes	yes
Lavebratt, 2019	February-2023	FSG	yes	yes	yes
Lee, 2016	March-2023	MPS	yes	yes	yes
Leke, 2018	February-2023	FSG	yes	yes	yes
Leong, 2020	March-2023	MPS	yes	yes	yes
Lin, 2020	March-2023	TSD	yes	yes	yes
Loewen, 2018	March-2023	MPS	yes	yes	yes
Lovern, 2022	February-2023	FSG	yes	yes	yes
Marild, 2014	March-2023	TSD	yes	yes	yes
Marild, 2017	February-2023	FSG	yes	yes	yes
Meeraus, 2015	February-2023	FSG	yes	yes	yes
Metzler, 2019	March-2023	MPS	yes	yes	yes
Miller, 2013	February-2023	FSG	yes	yes	yes
Miller, 2018	February-2023	FSG	yes	yes	yes

Supplementary Table 5. Continued. Data extraction summary and eligibility confirmation of included studies (n=79).

Mission, 2019	March-2023	MPS	yes	yes	yes
Mølgaard-Nielsen, 2012	February-2023	FSG	yes	yes	yes
Momen, 2015	February-2023	FSG	yes	yes	yes
Momen, 2021	March-2023	MPS	yes	yes	yes
Mor, 2015	March-2023	MPS	yes	yes	yes
Mubanga, 2021	February-2023	FSG	yes	yes	yes
Mueller, 2017	February-2023	FSG	yes	yes	yes
Nguyen, 2022	February-2023	FSG	yes	yes	yes
Nishigori, 2017	March-2023	TSD	yes	yes	yes
Olesen, 2006	March-2023	MPS	yes	yes	yes
Ortqvist, 2014	February-2023	FSG	yes	yes	yes
Petersen, 2010	March-2023	MPS	yes	yes	yes
Pisa, 2015	February-2023	FSG	yes	yes	yes
Rantala, 2022	February-2023	FSG	yes	yes	yes
Romanese, 2018	March-2023	TSD	yes	yes	yes
Rozanska, 2021	March-2023	MPS	yes	yes	yes
Sassonker-Joseph, 2021	March-2023	TSD	yes	yes	yes
Snyder, 2021	March-2023	MPS	yes	yes	yes
Stephansson, 2011	March-2023	MPS	yes	yes	yes
Stokholm, 2013	March-2023	TSD	yes	yes	yes
Stokholm, 2014	March-2023	TSD	yes	yes	yes
Tomar, 2022	March-2023	TSD	yes	yes	yes
Trinh, 2021	March-2023	TSD	yes	yes	yes
Turi, 2021	February-2023	FSG	yes	yes	yes
Uldbjerg, 2021	March-2023	MPS	yes	yes	yes
Valent, 2014	March-2023	TSD	yes	yes	yes
Wang, 2018	February-2023	FSG	yes	yes	yes
Ye, 2019	March-2023	MPS	yes	yes	yes
Yoshida, 2018	March-2023	MPS	yes	yes	yes
Zhang, 2019	March-2023	MPS	yes	yes	yes
Zhao, 2021	February-2023	FSG	yes	yes	yes
