# Supplementary material for: Prevalence of systemic antibacterial use during pregnancy worldwide: A systematic review
Source: PLoS One. 2024 Sep 6;19(9):e0309710. doi: 10.1371/journal.pone.0309710 (PMC11379220; doi:10.1371/journal.pone.0309710)
Supplement: Attachment — Submitted filename: Response to Reviewers.pdf [file pone.0309710.s007.pdf]

REBUTTAL LETTER

Manuscript ID: PONE-D-23-25313

Prevalence of systemic antibacterial use during pregnancy worldwide: a systematic review

We appreciate the thorough evaluation of our work by the two reviewers. Responses to each of the reviewers' comments are preceded by the word "**Response**". We believe the modifications made based on these comments have substantially improved the manuscript. Furthermore, we are at your disposal for further questions, doubts, or any clarification needed.

Reviewer #1

The review highlighted a wide range of antibiotic usage during pregnancy, with prevalence rates ranging from as low as 2% to as high as 64.3%. Notably, in low- and middle-income countries (LMICs), antibiotic use during pregnancy was observed to be between 2% and 48.3%, while outpatient primary care settings in LMICs showed prevalence rates spanning from 19.6% to 90.8%. Additionally, the review shed light on the challenges encountered in conducting this analysis, particularly in terms of limitations associated with the available data and the lack of comprehensive information provided by the included studies.

In my perspective, this review offers valuable insights, particularly regarding the epidemiology of antibiotic use during pregnancies.

Nevertheless, there are several areas where additional information could enhance our understanding. Firstly, it would be beneficial to examine the prevalence of antibiotic use both before and after the implementation of the WHO's AMR global plan. Additionally, investigating the common indications for antibacterial usage and stratifying prevalence based on these indications could provide valuable context.

Response: Thank you for your comments. We recognize the importance of the suggested analyses. The WHO's AMR global plan was published in 2016 and implemented in 2017. We identified only three studies that have performed data collection since 2016. The same occurred for included papers with information about indications, where we have

identified only six included studies. Unfortunately, there aren’t enough studies to perform these analyses.

Moreover, among the 79 included studies, it would be informative to categorize the types of antibiotics used, potentially utilizing the WHO's classification and categories for a more comprehensive analysis. Furthermore, Table 2 presents an opportunity to explore the prevalence of antibiotic use during different trimesters of pregnancy, allowing for a nuanced stratification and summary of this data. Addressing these aspects could contribute to a more comprehensive understanding of antibiotic usage patterns during pregnancy.

Response: Thank you for your comment. We identified 24 studies (representing 30% of the included studies) with information regarding the antibacterial classes. Therefore, we performed a descriptive analysis to categorize the subgroups of antibiotics used (new S3 Table). We included the following text in the Methods section: “*Information on the proportion of antibacterial subgroups of studies included is described in S3 Table. The number and proportion of the following subgroups were summarized: beta-lactam; sulphonamides and trimethoprim; macrolides, lincosamides and streptogramins; tetracyclines; quinolones; nitrofuran derivatives and imidazole*.”

Although stratification by trimesters of pregnancy could improve the comprehensiveness of the analysis, we identified only three studies with information on antibacterial use by trimesters. So, these analyses were not carried out due to the small number of studies with the requested information. We included the following text in the first paragraph of the Discussion section: “*We identified only three included studies with data on antibacterial use by trimesters (S2 Table). Unfortunately, we were unable to perform a comprehensive analysis due to the small number of studies with this information*.”

Other comments

Methodology:

• How about studies reporting in different languages? How did the authors process or analyse the article? Any translation done?

Response: We clarified the following information in Results section: “*All included studies were published in English language*.”

• How did the CI obtain? Was it from the value reported in the included studies or any additional analysis done?

Response: We clarified the following information in the Methods section: "*Confidence intervals (CI) were presented when reported by the authors of included studies*."

• In discussion, what does authors mean by response rate?

Response: We are now presenting the definition of response rate in the Methods section: "*Report response rates (i.e., proportion of pregnant women who answered the survey divided by the number of eligible pregnant women) in a flowchart or text.*"

Reviewer #2: The authors present an impressive exercise that attempts to generate estimates of for the prevalence of systemic antibacterial use during pregnancy. As a reviewer, I recognize the public health impact of this question, given different global initiatives to address antibiotic resistance. The manuscript is well organized, and the authors' objective is clear. The manuscript could benefit from clarification in several sections. Specifically, the authors discuss different biases in the use of primary vs secondary data, 1) without providing information how these biases would affect prevalence estimates and 2) that may not be relevant in the context of these analyses. There are a few sentences that could be rewritten to clarify meaning and improve reader comprehension. My comments and questions are the following:

Response: Thank you for your comments. We considerably improved the paragraph regarding primary and secondary studies. The following text preceded the discussion about measurement errors: "*Therefore, both errors could underestimate the prevalence of antibacterial use, although secondary studies with high-quality data may provide more reliable prevalence estimates, compared to primary studies*." Also, we added the likely effect of the LMP estimative to underestimate the antibacterial use during pregnancy, regarding secondary data studies: "*Additionally, the pregnancy period misclassification resulting from LMP inaccuracy is greater for short-term use characteristic of antibacterials, compared to medicines used to treat chronic conditions, which can probably underestimate the prevalence of antibacterial use during pregnancy*."

Comprehension or interpretation-based questions or comments:

1. The authors exclude case-control studies on the basis that samples included in case-control studies are non-representative. However, based on the sampling scheme of a case-control study, the controls represent the underlying exposure distribution in the population. Since many studies examining use of medications during pregnancy are case-control studies (particularly comparative studies of exposure to medications during pregnancy and rare outcomes such as congenital anomalies), are the authors concerned that they may be missing crucial information to inform prevalence estimates?

Response: We appreciate your very interesting thoughts about case-control studies. However, we were interested in population-based measures, and control groups could be non-representative. Additionally, depending on the outcome, the control group may be associated with antibacterial use during pregnancy, making it challenging to imply

representativeness. Thus, the selection of controls is one of the main sources of bias concerning case-control design. We added the following text regarding the limitations: "*Fourth, we did not include search terms related to case-control studies. We opted for population-based cohort and cross-sectional studies to summarize the prevalence, compared to nested case-control studies, due to the selection bias that may ensue in case-control designs*."

2. There were 18,418 titles and abstracts screened and of those, 164 were assessed for eligibility. The authors should describe the reason(s) fewer than 10% of the titles and abstracts screened were assessed for eligibility, especially since they made no exclusions for time, date or language in their search terms. For example, did the authors exclude other systematic reviews on the prevalence antibiotic use during pregnancy (e.g., Kuperman AA, Koren O. Antibiotic use during pregnancy: how bad is it? BMC Med. 2016;14(1):91. Published 2016 Jun 17. doi:10.1186/s12916-016-0636-0)?

Response: We thank you for your comment. We performed a comprehensive search, and the majority of studies were about antimicrobial resistance, medicine use, and drug use in pregnancy. Also, we found epidemiologic antenatal studies and protocols. In cases of reviews and systematic reviews, the references were searched (considering the paper aforementioned, for example). However, we did not have the exact number of published reviews since we've had 18,418 papers screened for titles and abstracts. Finally, the paper from Kuperman and Koren did not present a systematic search (i.e., clearly defined criteria for database search, time period, and search terms, with the paper's eligibility criteria), therefore it can't be classified as a systematic review.

We added the information about how we processed this issue in the Methods section: "*Additionally, we scanned the references of reviews and systematic reviews given by the search strategy.*". We included the terms *reviews and systematic reviews* on the ineligible studies paragraph. Also, we included the following sentence in the Results section: "*We excluded 18,247 records for reasons such as study type according to exclusion criteria and theme out of scope, resulting in 164 records assessed for eligibility, leading to 77 included studies*"

3. The authors include a through line at ~28% prevalence in Figure 3. Is there a particular reason for this? It wasn't clear to the reviewer when reading the methods section

Response: Thank you for your comment. The line represents the average prevalence estimate. We clarified in the text and table footnote.

4. The authors mention in the discussion section that "there is a chance of underestimation regarding primary data study estimates compared to secondary dataset studies, and these need to be properly interpreted. The last one is recorded prospectively and independently, avoiding maternal recall bias". First, this sentence is difficult to understand. When stating the "last one" are the authors referring to the former (primary data) or the latter (secondary data)? Second, maternal recall bias is not simply a result of retrospective data collection,

but rather a particular scenario was recall of the exposure is differential by the outcome. Thus, it is unclear how simply using secondary data would lead to recall bias in this case. It may lead to recall error but not bias.

Response: We appreciate your comment. We have made the clarifications in this paragraph. First, we rewrote the sentence referring to secondary data: "*The latter is recorded prospectively and independently, which can help to minimize maternal recall bias. Additionally, well designed secondary data studies depend on the quality of the data, while primary studies rely on the recall period and the research question*." Second, the absence of data can occur in both types of studies (primary and secondary data), since the report of antibacterial use during pregnancy could rely on the questionnaire and recall period for primary studies and lack of completeness regarding secondary data studies, depending on data quality. We added the following text: "*Depending on the type of data, measurement errors can result from different sources: 1) The absence of data for prescription database studies; and 2) The lack of reporting the medicine use collected from interviews or self-administered questionnaires in primary data studies. Therefore, both errors could underestimate the prevalence of antibacterial use, although secondary studies with high-quality data may provide more reliable prevalence estimates compared to primary studies.*"

5. The authors state that studies using both primary and secondary data sources have to deal with misclassification, since prescription and dispensing information may not reflect actual use. Again, the authors seem to be conflating terms that may lead to confusion for the reader. Since the authors present a descriptive analysis rather than a comparative one (with association or effect measures), it seems more likely that they are referring to measurement error rather than bias. If the authors do in fact believe that there would be bias (instead of simply error), they should be specific about the type of misclassification that would occur in these scenarios. For example, in a prospective study, they are more likely to incur non-differential misclassification of the exposure (i.e., use of antibacterials during pregnancy).

Response: Thank you for your comment. We clarified the different sources of misclassification in the discussion section as shown above, considering the lack of quality in secondary data and the use of antibacterial consumption *per se*. Bias can be defined as the result of a systematic error in the design or conduct of a study. Therefore, we have to deal with two levels of errors that could imply bias: The first is related to the lack of data from the secondary database, mainly in LMICs, and the absence of antibacterial use reports from primary studies. The second level has to do with the antibacterial consumption by pregnant women, because the number of antibacterial prescriptions may not accurately reflect the amount of antibacterial dispensed or the actual use.

6. The authors state that differences in the range of prevalence estimates may be due to differences in calculating gestational age. While that may certainly play a role, the authors should also specifically address how the different exposure windows may affect prevalence estimates. Evaluating prevalence of use of antibacterials during the 1st, 2nd

and 3rd trimester, is predicated on pregnancies continuing for longer gestational periods and thus have a greater opportunity to be exposed to antibiotics (compared than pregnancies that result in early losses or preterm delivery).

Response: We agree with the comment. However, we did not take into account these differences, and we considered studies with complete information (1st, 2nd, and 3rd trimesters) about the use of antibiotics in pregnancy. We added the following text in the LMP paragraph: "*All studies reported the overall prevalence of antibacterial use throughout the pregnancy period (1st, 2nd, and 3rd trimesters). In general, information on induced terminations and spontaneous pregnancy losses is available in secondary databases [102]. This data can be considered too sensitive to be publicly released for research [102] and may lead to differences in exposure to antibacterials in pregnancy*"

7. In the discussion section, the authors state the "there is a chance of under-detection bias by antibacterial over-the-counter (OTC) sales" when discussion a study using data from the Tennessee Medicaid Program. As the reviewer is aware, there are no systemic antibacterials that are approved or available for OTC dispensing in the US.

Response: Thank you for your comment. In this paragraph, we aimed to discuss insurance and sales databases, and we corrected the text: "*The highest prevalence of 64.3% (95%CI not reported) in the overall sample was reported from an American study of mother-child dyads with data from the insurance database Tennessee Medicaid Program (TennCare) [29]. We identified studies using insurance, reimbursement, and sales databases. Importantly, the use of sales databases, compared to insurance databases, raises multiple challenges*."

8. In the discussion section, the authors state "since the database may not cover a large segment of the population not covered, given the lack of data from the public sector", again referring to data from Tennessee Medicaid Program. As the reviewer is aware, Medicaid is a joint federal and state program which gives health coverage to people with limited income and would be considered the public coverage option.

Response: Thanks for your commentaries about the Tennessee Medicaid Program. We reformulated the text, specifying the comparison among prescription and sales databases: "*Second, chance of data collection bias since the sales database may not cover a large segment of the population not covered, given the lack of data from public sector.*"

Minor/Editorial comments

1. In the introduction, the authors write that the inappropriate use of antibacterial can lead to antibacterial resistance (AMR). AMR refers to antimicrobial resistance (resistance to antimicrobials, includes resistance to antivirals, antifungals and antiparasitics, in addition to antibacterials while antibacterial resistance only refers to resistance to the latter). Please correct this in the manuscript

Response: We made the correction. Thank you.

2. Please remove the article “The” before “The AMR” in the second sentence of the second paragraph in the introduction

Response: Done.

3. Please consider removing the article numbers in the text of the results section when describing the proportion of articles that provided information on various characteristics (e.g., maternal age). For example, the sentence “regarding the 79 included studies, 43 provided information on maternal age [listed article numbers]” should have the listed article numbers removed. The authors may consider simply referencing table S2

Response: Thank you for your comment. We opted for the article numbers in order to facilitate the reader’s comprehension. However, we are flexible with the editor’s decision.
