# Supplementary material for: Prevalence of systemic antibacterial use during pregnancy worldwide: A systematic review
Source: PLoS One. 2024 Sep 6;19(9):e0309710. doi: 10.1371/journal.pone.0309710 (PMC11379220; doi:10.1371/journal.pone.0309710)
Supplement: Attachment — Submitted filename: Response to Reviewers.pdf [file pone.0309710.s008.pdf]

REBUTTAL LETTER

Manuscript ID: PONE-D-23-25313R1

Prevalence of systemic antibacterial use during pregnancy worldwide: a systematic review

We appreciate the thorough evaluation of our work by the reviewers. The word "**Response**" precedes each response to the comments. We believe the modifications made based on these comments have substantially improved the manuscript. Furthermore, we are available to answer any additional questions, doubts, or clarifications you may have.

Reviewer #1: The revised version of the manuscript did not respond to my previous comments. In addition, there was no response to each of the comments.

Response: Dear reviewer, considering the commentary on the manuscript and the response letter from the first round of revision, we thoroughly examined the response letter to identify any unanswered questions. Additionally, we reviewed the manuscript's text to ensure the response letter aligns with the revised version.

Our answers may have been justifications for not following some recommendations, as in the case of the first presented comment: *"Firstly, it would be beneficial to examine the prevalence of antibiotic use both before and after the implementation of the WHO's AMR global plan. Additionally, investigating the common indications for antibacterial usage and stratifying prevalence based on these indications could provide valuable context."*

In light of the second review, we have revised our response to this commentary. We included analyses to consider the first suggestions, which we initially decided not to perform due to the small number of studies in the resulting subgroups. In this second-reviewed version, we describe the prevalence of antibacterial use before and after the WHO's AMR global plan was implemented. We included the following text in the Results section: "*Subsequently to the Global Action Plan on Antibacterial Resistance stated by WHO in 2016 [8], only three studies [28,52,85] were performed using data collection since 2016, and the proportions ranged from 2.6% (95%CI not reported) to 8.2% (95%CI not reported). Studies with data collected before 2016 showed a prevalence ranging from 2.0% (95%CI 2.0–2.0) to 64.3% (95%CI not reported).*" Similarly, we identified only six studies with information about indications, and we reported the most common indications

in the Results section, as follows: "*Only six studies [11,23,25,28,47,81] provided information on antibacterial indications. Among those, the most common indications were urinary tract infections and respiratory tract infections*.". We reported both analyses in the Methods section: "*We described proportions of antibacterial use during pregnancy before and after the Global Action Plan on Antibacterial Resistance stated by the WHO. We identified the most common indications for antibacterial use during pregnancy among the included studies*." Unfortunately, additional analyses were not feasible due to the small number of studies. Finally, we reviewed all comments and answers from the first round of revisions. Any further unclear points or concerns identified by the reviewer will be addressed. We would be pleased to revisit and make additional adjustments to the text as necessary.

Reviewer #1: In the current version, while the introduction provides valuable context and highlights important issues related to antibacterial use during pregnancy and antimicrobial resistance (AMR), it does not sufficiently justify the specific objectives of the study. It would benefit from a clearer linkage between the background information and the study's aims. Specifically, it should better articulate why it is important to perform a descriptive analysis of study-level characteristics. Additionally, it should explain how these findings will contribute to addressing the gaps in knowledge, particularly in low- and middle-income countries (LMICs) as the results also included non-LMICs

Response: Thank you for your comment. We underlined the importance of the descriptive analysis of study-level characteristics, including the following text in the introduction section: *A tabular summary of prevalence point estimates can provide useful information of evidence synthesis [12]. Thus, gathering data on the proportion of antibacterial use during pregnancy enables the comparability between subgroups of studies, in order to identify point estimates variability according to relevant characteristics, such as study type, data source and country income"*. Also, we added in the introduction section information about the relevance of the results focused on LMICs: "*Despite this difference, estimates from LMICs can provide an overview of the likely prevalence of antibacterial use during pregnancy, addressing the needs of the AMR action plans in LMICs [6,8]. Further information on trends of antibacterial use and indication is also needed."*

Reviewer #1: The resolution of Figure 1 to 4 are poor. very hard to read

Response: As suggested, we improved the resolution of figures using the Preflight Analysis and Conversion Engine (PACE) digital diagnostic tool.
